# Breaking boundaries: a novel role for *CUC* genes in sex determination in cucurbits

**DOI:** 10.1093/jxb/erae056

**Published:** 2024-03-27

**Authors:** Margaret Anne Pelayo, Frank Wellmer

**Affiliations:** Department of Genetics, Trinity College Dublin, Dublin, Ireland; Department of Genetics, Trinity College Dublin, Dublin, Ireland

**Keywords:** Boundary specification, *CUC2*, *Cucurbita pepo*, ethylene, female flowering, miR164

## Abstract

This article comments on:

Segura M, García A, Gamarra G, Benítez A, Iglesias-Moya J, Martínez C, Jamilena M. 2024. An *miR164*-resistant mutation in the transcription factor gene *CpCUC2B* enhances carpel arrest and ectopic boundary specification in *Cucurbita pepo* flower development. Journal of Experimental Botany 75, 1948–1966.

**Elucidating the mechanisms underlying sex determination in flowers is not only essential for a better understanding of plant reproductive biology but it also has important agronomic implications as it underpins crop breeding efforts. In this issue, Segura *et al.* (2024) show that in *Cucurbita pepo*, the NAC-family transcription factor-coding gene *CUP-SHAPED COTYLEDON 2B* (*CpCUC2B*), which is post-transcriptionally regulated via the conserved miR164 miRNA pathway, is a critical determinant for sex.**

Plant sexual systems are highly diverse and have long been a fascinating subject of study among biologists (Harkess and Leebens-Mack, 2017). Most angiosperms, or flowering plants, are hermaphrodites wherein both male and female organs are present in a single individual (Bachtrog *et al.*, 2014). In this case, hermaphroditism can take a broad range of forms, from the classic ‘perfect’ flower with functional male and female organs found within single flowers of an individual (bisexual flower), to monoecy with separate male and female flowers occurring on an individual plant (unisexual flowers) (Dellaporta and Calderon-Urrea, 1993; Pannell, 2017). Gynodioecy and androdioecy are also forms of plant hermaphroditism where individuals produce hermaphroditic and/or only female or only male flowers, respectively (Masuda and Akagi, 2023). Dioecy, or the development of individual male and female plants, is considered to have evolved from hermaphroditism. In fact, it is generally considered that all current forms of sexual expression in flowering plants evolved from an ancestral hermaphroditic progenitor and that distinct sexes evolved repeatedly and only relatively recently (Dellaporta and Calderon-Urrea, 1993; Charlesworth, 2002; Bachtrog *et al.*, 2014; Masuda and Akagi, 2023). This could be seen as in direct contrast to well-established sex determination mechanisms among animals, particularly in mammals, wherein sexuality can be attributed solely to X and Y sex chromosomes (XX for females and XY for males) derived from a master-switch gene (*Sry*) in ancestral autosomes (Foster and Graves, 1994; Bachtrog *et al.*, 2014). Thus, sexual determinacy is more variable and fluid for plants when compared with other organisms (Tanurdzic and Banks, 2004; Diggle *et al.*, 2011; Bachtrog *et al.*, 2014).

## Arrested development: monoecious sex determination in *C. pepo*

**Fig. 1.**
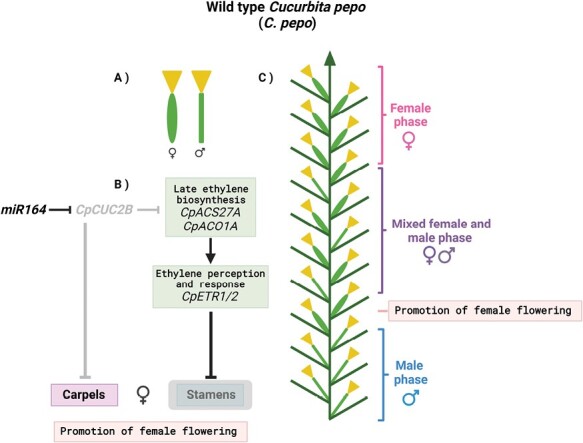
Sexual development in wild-type (WT) *Cucurbita pepo*. (A) Graphical representation of the unisexual flowers of *C. pepo*. The calyx is represented by the inverted yellow triangle. Female and male flowers are distinguished by the shape of their peduncles (green). Female flowers have an elongated oval peduncle while male flowers have a straight and slender peduncle. (B) *CpCUC2B* is upstream of the late ethylene biosynthesis and response pathway. *CpCUC2B* expression is post-transcriptionally regulated by miR164 and suppresses female flower development. *CpCUC2B* also probably acts synergistically with the ethylene perception and response genes *CpETR1/2* for overall sex determination. (C) Graphical representation of *C. pepo* sexual developmental phases (read from bottom to top). Sexual development is preceded by an initial male phase (blue bracket and text) where only male flowers are formed. The phase when female flowers first form is considered as the moment when the promotion of female flowering commences (boxed text in pink). The developmental events initiated by the regulatory pathway in (B) probably occur during this phase. From this point, a mixed male and female phase (purple bracket and text) proceeds until the final phase featuring mostly, or exclusively, female flowers, considered as the female phase (pink bracket and text). Figure generated using BioRender.

Various species have emerged as models for elucidating the control of sex determination in plants, such as white campion (*Silene latifolia*) and sorrel (*Rumex*) where plant sex chromosomes were initially discovered from as early as 1923 (Westergaard, 1958; Harkess and Leebens-Mack, 2017; Masuda and Akagi, 2023). More recently, various agronomically valuable species have also made this list, including melon (*Cucumis melo*), papaya (*Carica papaya*), strawberry (*Fragaria* spp.), persimmon (*Diospyros lotus*), and garden asparagus (*Asparagus officinalis*), to name a few (Byers *et al.*, 1972; Boualem *et al.*, 2008; Spigler *et al.*, 2008; Yu *et al.*, 2008; Akagi *et al.*, 2014; Harkess *et al.*, 2015; Harkess and Leebens-Mack, 2017; Zhang *et al.*, 2022). Apart from melon, other members of the *Cucurbitaceae* family are also widely used in sex determination studies, including *C. pepo* which encompasses the agriculturally important squash, pumpkin, and zucchini morphotypes (Martínez and Jamilena, 2021). *Cucurbita pepo* is a monoecious member of the *Cucurbitaceae* that exhibits three distinct stages of sexual development (Fig. 1C). Upon reaching reproductive maturity, it goes through an initial male phase, producing only unisexual male flowers. This is followed by a hermaphroditic mixed phase with male and female flowers alternately produced. The final stage is a female phase comprising a majority of, or exclusively, unisexual female flowers (Schilling *et al.*, 2020; Martínez and Jamilena, 2021; Segura *et al.*, 2024). For monoecious plants such as *C. pepo*, this type of sex determination mechanism can be considered as ‘developmental’ wherein sexuality is not specifically controlled by sex chromosomes but rather through the activity of various regulatory factors leading to the arrest of either carpel development to promote the male phase, or stamen development to promote the female phase (Pannell, 2017; Martínez and Jamilena, 2021). Among those regulatory factors, the phytohormone ethylene plays a central role as it has been demonstrated that ethylene biosynthesis and signalling determine female development across cucurbits (Pannell, 2017; Schilling *et al.*, 2020; Martínez and Jamilena, 2021).

## Masters of sex: transcription factors, miRNA, and hormones as regulators of sex determination

**Fig. 2.**
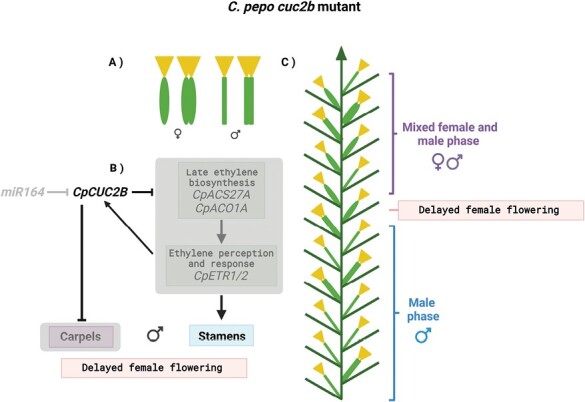
Sexual development in the *C. pepo cuc2b* mutant. (A) Graphical representation of the unisexual flowers in *cuc2b*. Wild-type flowers develop along with double flowers exhibiting a fused calyx (inverted yellow triangles) and fused peduncles (green oval or elongated bar structure) phenotypes for both the female and male flowers. (B) *cuc2b* is a gain-of-function mutation that suppresses the late ethylene pathway produced by *CpACS27* and *CpACO1A*, so promoting the development of stamens which are inactive as miR164 is unable to post-transcriptionally repress *CpCUC2B*. (C) Graphical representation of *cuc2b* sexual developmental phases (read from bottom to top). In *cuc2b*, the male phase is extended as a result of a delayed female flowering transition. The mixed female phase is also delayed, and the overall result is a nearly androecious phenotype with a reduced number of female flowers. Both the wild-type and fused flowers are depicted here. Figure generated using BioRender.

In this issue, the study by Segura *et al.* (2024) introduces the *C. pepo* NAC-family transcription factor-coding gene *CUP-SHAPED COTYLEDON 2B* (*CpCUC2B*) as a new transcriptional regulator involved in the sex determination pathway in zucchini. *CpCUC2B* is a homologue of the *Arabidopsis thaliana CUC2* gene that is well known for specifying and defining organ boundaries along with *CUC1* and *CUC3* during embryogenesis, shoot apical meristem formation, flower development, and leaf development (Mallory *et al.*, 2004; Maugarny *et al.*, 2016). The current study by Segura and colleagues is the first description of *CUC* genes playing a role in sex determination, indicating that *CUC* gene functions in other plant families can expand and diverge to include control of reproductive development. Using a forward genetics approach, the group initially identified the semi-dominant *cuc2b* mutant (Fig. 2C). *cuc2b* plants display mostly male flowers as a result of a prolonged male phase and delayed female flowering. Flower development was also altered in *cuc2b* mutants, namely during floral meristem specification resulting in increased floral organ numbers and fused floral organ phenotypes (Figs 1A, 2A). The authors showed that the causative mutation in *cuc2b* is located in exon 3 of *CpCUC2B*. Notably, they found that this mutation is in the binding site of members of the miR164 family of miRNAs, which are known to post-transcriptionally regulate NAC-domain genes, including Arabidopsis *CUC2* (Mallory *et al.*, 2004; Sieber *et al.*, 2007). In Arabidopsis, it has been shown that the miR164-dependent regulation fine-tunes *CUC* gene expression and is essential for proper organ boundary establishment and formation (Laufs *et al.*, 2004; Nikovics *et al.*, 2006; Adam *et al.*, 2011). In *C. pepo*, the mutation in the miR164-binding site renders *CpCUC2B* resistant to miR164 regulation, leading to a gain of *CpCUC2B* function. The observed nearly androecious *C. pepo* phenotype in *cuc2b* therefore suggests that *CpCUC2B* promotes male flowering by arresting carpel development (Figs 1B, 2B). The results of additional experiments, in which double mutants between *cuc2b* and ethylene biosynthesis or signalling mutants were analysed, further imply that *CpCUC2B* is also involved in suppressing the arrest of stamen development and that the interaction of *CUC2B* with the ethylene response pathway is complex and probably differs in male and female reproductive organs. Overall, the results suggest that the interplay between ethylene, transcription factors, and miRNA-mediated post-transcriptional pathways is a key determinant of monoecious sex determination.

## Moving beyond limits: understanding plant sex determination for better plant productivity and resilience

Uncovering the genetic and molecular mechanisms underlying sex determination in flowering plants is essential for a comprehensive understanding of how sex evolved throughout living systems. No less important and profound is this knowledge for how we can better adapt plants, especially those that we rely on for sustenance and livelihood (such as many *C. pepo* morphotypes), in a world with increasingly adverse climatic conditions and food insecurity. The work by Segura and colleagues adds a novel factor in the current sex determination framework for cucurbits that will form the basis for future activities aimed at improving plant productivity and resilience. Strategies in improving crop pollination success, yield, and fruit quality, and implementing cropping and breeding approaches are just some examples in our agricultural systems that would stand to benefit from similar advances in plant sex determination studies (Masuda and Akagi, 2023). To understand how sex determination and overall reproductive success are affected by abiotic and biotic stresses will also be of significant interest in the face of erratic weather events brought on by climate change and in improving crop productivity to keep pace with unprecedented global population growth.

## References

[CIT0001] Adam H , Marguerettaz M, Qadri R, et al. 2011. Divergent expression patterns of miR164 and CUP-SHAPED COTYLEDON genes in palms and other monocots: implication for the evolution of meristem function in angiosperms. Molecular Biology and Evolution 28, 1439–1454. 21135149 10.1093/molbev/msq328

[CIT0002] Akagi T , Henry IM, Tao R, Comai L. 2014. A Y-chromosome-encoded small RNA acts as a sex determinant in persimmons. Science 346, 646–650. 25359977 10.1126/science.1257225

[CIT0003] Bachtrog D , Mank JE, Peichel CL, et al. 2014. Sex determination: why so many ways of doing it? PLoS Biology 12, e1001899. 24983465 10.1371/journal.pbio.1001899 PMC4077654

[CIT0004] Boualem A , Fergany M, Fernandez R, et al. 2008. A conserved mutation in an ethylene biosynthesis enzyme leads to andromonoecy in melons. Science 321, 836–838. 18687965 10.1126/science.1159023

[CIT0005] Byers RE , Baker LR, Sell HM, Herner RC, Dilley DR. 1972. Ethylene: a natural regulator of sex expression of *Cucumis melo* L. Proceedings of the National Academy of Sciences , USA 69, 717–720. 10.1073/pnas.69.3.717 PMC426542 16591971

[CIT0006] Charlesworth D. 2002. Plant sex determination and sex chromosomes. Heredity 88, 94–101. 11932767 10.1038/sj.hdy.6800016

[CIT0007] Dellaporta SL , Calderon-Urrea A. 1993. Sex determination in flowering plants. The Plant Cell 5, 1241–1251. 8281039 10.1105/tpc.5.10.1241 PMC160357

[CIT0008] Diggle PK , Di Stilio VS, Gschwend AR, Golenberg EM, Moore RC, Russell JRW, Sinclair JP. 2011. Multiple developmental processes underlie sex differentiation in angiosperms. Trends in Genetics 27, 368–376. 21962972 10.1016/j.tig.2011.05.003

[CIT0009] Foster JW , Graves JA. 1994. An SRY-related sequence on the marsupial×chromosome: implications for the evolution of the mammalian testis-determining gene. Proceedings of the National Academy of Sciences, USA 91, 1927–1931. 10.1073/pnas.91.5.1927 PMC43277 8127908

[CIT0010] Harkess A , Leebens-Mack J. 2017. A century of sex determination in flowering plants. Journal of Heredity 108, 69–77. 27974487 10.1093/jhered/esw060

[CIT0011] Harkess A , Mercati F, Shan H-Y, Sunseri F, Falavigna A, Leebens-Mack J. 2015. Sex-biased gene expression in dioecious garden asparagus (*Asparagus officinalis*). The New Phytologist 207, 883–892. 25817071 10.1111/nph.13389

[CIT0012] Laufs P , Peaucelle A, Morin H, Traas J. 2004. MicroRNA regulation of the CUC genes is required for boundary size control in Arabidopsis meristems. Development 131, 4311–4322. 15294871 10.1242/dev.01320

[CIT0013] Mallory AC , Dugas DV, Bartel DP, Bartel B. 2004. MicroRNA regulation of NAC-domain targets is required for proper formation and separation of adjacent embryonic, vegetative, and floral organs. Current Biology 14, 1035–1046. 15202996 10.1016/j.cub.2004.06.022

[CIT0014] Martínez C , Jamilena M. 2021. To be a male or a female flower, a question of ethylene in cucurbits. Current Opinion in Plant Biology 59, 101981. 33517096 10.1016/j.pbi.2020.101981

[CIT0015] Masuda K , Akagi T. 2023. Evolution of sex in crops: recurrent scrap and rebuild. Breeding Science 73, 95–107. 37404348 10.1270/jsbbs.22082 PMC10316312

[CIT0016] Maugarny A , Gonçalves B, Arnaud N, Laufs P. 2016. CUC transcription factors: to the meristem and beyond. In: Gonzalez DH, ed. Plant transcription factors. Boston: Academic Press, 229–247.

[CIT0017] Nikovics K , Blein T, Peaucelle A, Ishida T, Morin H, Aida M, Laufs P. 2006. The balance between the MIR164A and CUC2 genes controls leaf margin serration in Arabidopsis. The Plant Cell 18, 2929–2945. 17098808 10.1105/tpc.106.045617 PMC1693934

[CIT0018] Pannell JR. 2017. Plant sex determination. Current Biology 27, R191–R197. 28267976 10.1016/j.cub.2017.01.052

[CIT0019] Schilling S , McCabe PF, Melzer R. 2020. Love is in the air: ethylene and sex determination in *Cucurbita pepo*. Journal of Experimental Botany 71, 4–6. 31602481 10.1093/jxb/erz412

[CIT0020] Segura M , García A, Gamarra G, Benítez A, Iglesias-Moya J, Martínez C, Jamilena M. 2024. An *miR164* resistant mutation in the transcription factor gene *CpCUC2B* enhances carpel arrest and ectopic boundary specification in *Cucurbita pepo* flower development. Journal of Experimental Botany 75, 1948–1966. 10.1093/jxb/erad486 PMC10967242 38066672

[CIT0021] Sieber P , Wellmer F, Gheyselinck J, Riechmann JL, Meyerowitz EM. 2007. Redundancy and specialization among plant microRNAs: role of the MIR164 family in developmental robustness. Development 134, 1051–1060. 17287247 10.1242/dev.02817

[CIT0022] Spigler RB , Lewers KS, Main DS, Ashman T-L. 2008. Genetic mapping of sex determination in a wild strawberry, *Fragaria virginiana*, reveals earliest form of sex chromosome. Heredity 101, 507–517. 18797475 10.1038/hdy.2008.100

[CIT0023] Tanurdzic M , Banks JA. 2004. Sex-determining mechanisms in land plants. The Plant Cell 16, S61–S71. 15084718 10.1105/tpc.016667 PMC2643385

[CIT0024] Westergaard M. 1958. The mechanism of sex determination in dioecious flowering plants. Advances in Genetics 9, 217–281. 13520443 10.1016/s0065-2660(08)60163-7

[CIT0025] Yu Q , Navajas-Pérez R, Tong E, Robertson J, Moore PH, Paterson AH, Ming R. 2008. Recent origin of dioecious and gynodioecious Y chromosomes in papaya. Tropical Plant Biology 1, 49–57.

[CIT0026] Zhang S , Tan F-Q, Chung C-H, et al. 2022. The control of carpel determinacy pathway leads to sex determination in cucurbits. Science 378, 543–549. 36378960 10.1126/science.add4250

